# Financial Disruption and Psychological Underpinning During COVID-19: A Review and Research Agenda

**DOI:** 10.3389/fpsyg.2022.878706

**Published:** 2022-07-14

**Authors:** Sanjeet Singh, Deepali Bedi

**Affiliations:** ^1^University Centre for Research & Development, Chandigarh University, Mohali, India; ^2^University School of Business, Chandigarh University, Mohali, India

**Keywords:** pandemic, COVID-19, Corona, psychology, finance, stress, SDG, sustainability

## Abstract

Coronavirus disease 2019 (COVID-19) has disastrous impacts on sustainability initiatives and worsened poverty, hunger, and health issues. The financial distress by this pandemic has resulted in psychological challenges among the people. The list of vulnerable sections of the psychological impacts of the financial problems during COVID-19 is led by students, pregnant women, children, old age people, parents, and many more. The recommendations of this article are to focus on coping strategies for managing psychological issues related to financial problems during the pandemic, facilitating social support, promoting public health facilities and health insurance, financial support for pregnant women, and child care. Special care for old-age people and support for troubling parents and students. This article also recommends interventions and policies for reviving the disrupted businesses and strengthening entrepreneurs. Measures for income generation, removal of hunger, and reducing addictions and gambling should also be on the priority list. Limited collaboration among countries was observed, but robust collaboration among the research institutions and authors.

## Introduction

Global goals of sustainable development are going ahead with seventeen sustainable development goals (SDGs). Poverty, hunger, and health are focused on the first three SDGs. Coronavirus disease 2019 (COVID-19) has directly washed away the efforts on SDG achievements, more specifically related to poverty, hunger, and health-related goals. The negative impacts of the pandemic on these goals are yet to be completely assessed. The interim report on COVID-19 and SDG achievements (Guterres, 2020) shows the severity of the impact on income, wealth, hunger, and health. A total of 119–124 million people were added up to the extreme poverty and billions are waiting outside without any coverage of social security scheme. Similarly, 70–161 million people have faced hunger due to the pandemic. These facts point out the huge threat, the world is facing on undernutrition, stunted growth, and wasting. The erosion of achievements in reproductive, child, and maternal healthcare is a reality (Guterres, 2020). This works as a vicious cycle and financial distress and psychological stress can be dangerous during COVID-19. The COVID-19 has caused huge disruption in the global economy and affected the income and wealth of people and the state. The day-to-day expenditures have increased and income sources have been dried up. This resulted in the erosion of wealth. But what can be done with the financial and job requirements of millions without any stable income and significant wealth during the pandemic? COVID-19 has also impacted several businesses and job losses. This disruption has caused several psychological impacts on people, especially related to financial wellbeing.

Coronavirus disease 2019 (COVID-19)-related travel and social life restrictions, especially the long lockdowns had stopped the income pipelines of millions. All the businesses, except the essential goods, were closed during the first wave of the COVID-19. Even the essential good businesses were allowed to operate on selected days and restrictions on the operation time. COVID-19 impacted the global economies (Nelson et al., 2020) and there are very few economies with positive GDP growth in 2020–2021. Along with the financial distress, the anxiety about job, future, and the spread of the novel Coronavirus has challenged the mental health of the majority of the global populations by one way or the other. A major portion of the global population is without financial and job security and is outside the brackets of social security schemes and medical security like health insurance, and all this builds ups the psychological pressure related to financial wellbeing in the individuals.

The COVID-19 has a multidimensional impact on people, especially related to the psychological linkages with financial wellbeing. There is evidence for the financial stress and the linkages among diversified cross-sections of the population, especially the older sections (Lee et al., 2022). People are facing financial fragility during COVID-19, and financial confidence through financial literacy can contain the negatives of the financial fragility during the pandemic (Chhatwani and Mishra, 2021). The COVID-19 has affected the work and life of workers including the healthcare workers like lab nurses and technologists, causing financial stress and psychological pressures due to the relocation, lay-offs, furloughs, and diminished work hours (Ann Estes et al., 2021). COVID-19 has also an impact on the student’s psychological health, related to their financial status and academic performances. The mental health of students can play a significant role in handling the psychological issues related to COVID-19 and financial status, and for better academic performances (Kokkinos et al., 2022). Care should be taken that the stress related to COVID and threats to financial wellbeing can increase the chances for negative addictions including alcohol (Lechner et al., 2021; Rodriguez et al., 2021). The psychological linkages of the risk in financial wellbeing during COVID can have significant impacts on the children. Research has identified the linkages between financial wellbeing and the mental health of children during the pandemic (Mâsse et al., 2021). The self-employed populations are one of the vulnerable sections of society, facing short-term psychological stress due to the financial challenges associated with COVID-19 (Patel and Rietveld, 2020). The major front-line workers like health workers and retailers were one of the most vulnerable to psychological and financial stress during the COVID-19 pandemic and the financial service workers are also not free from the psychological stress during the pandemic. The experiences of psychological challenges during COVID-19 among financial services workers in Bangladesh are a very good example in this regard. However, the main concern for stress in this section is related to the spreading of the virus and health-related, rather than the concerns on financial wellbeing (Rana and Islam, 2021).

The literature on the psychological pinning’s on the financial wellbeing of people is very limited in the literature, especially the reviews and bibliometric analysis. This article includes the thematic analysis on this topic by adopting the systematic literature review. The bibliometric patterns of the literature are also included to cover the gap in the literature related to the psychological impacts on financial wellbeing during COVID-19. The study used the PRISMA guidelines for the selection of the articles and structure of the study. This section of the introduction is followed by methodology discussion, sections of thematic and bibliometric discussions, and followed by units for discussing the research gap and conclusion.

### Review Objectives

1. To study the financial wellbeing and psychological linkages during COVID-19
2. To summarize the literature on financial wellbeing and psychological linkages during COVID-19 from the resources of Web of Science
3. To develop future agenda for research on financial wellbeing and psychological linkages during COVID-19

### Research Questions

1. What are the psychological connections of financial wellbeing during the COVID-19?
2. What are psychological determinants associated with financial wellbeing, during the COVID-19?
3. What are the bibliometric patterns on the topic of financial wellbeing and psychological linkages during COVID-19?
4. What is the future agenda for research and recommendations on the topic of psychological connections of financial wellbeing during the COVID-19?

## Review Methodology

This review on psychological connections on financial wellbeing during the COVID pandemic has used the Web of Science database for developing bibliometric and thematic analysis. The resource platform of Web of Science is professionally managed to have more than 79 million records, in a multidisciplinary fashion. In contradiction to the practice of using multiple databases for systematic review, this article has adopted a single-source model, drawing motivation from the high-quality literature with the single-source model (Lage Junior and Godinho Filho, 2010; Jabbour, 2013; Talan and Sharma, 2019). This review article has used topic function (TS), and used the Boolean “psychological financial COVID wellbeing.” The documents for this review were downloaded on 11 February 2022.

**FIGURE 1:**
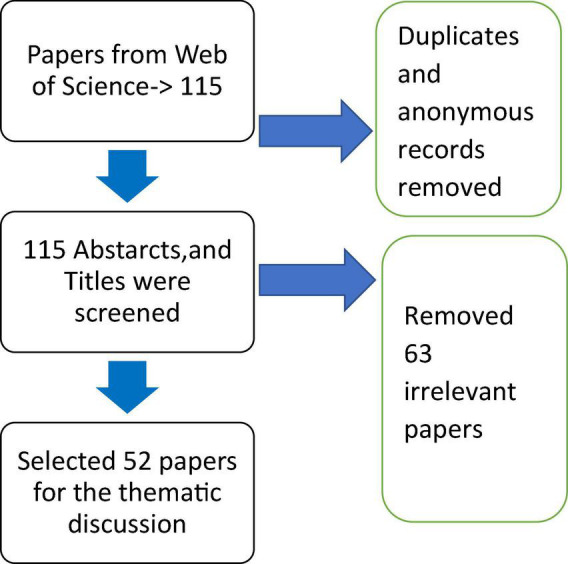
Paper identification and screening process.

The duplicates and anonymous records were removed and obtained 115 documents. All types of documents were used for both thematic and bibliometric analyses. All 115 papers are used for the bibliometric analysis and 53 papers are used for the thematic analysis. This review followed PRISMA guidelines along with the details of the article acceptance and rejection, shown in Figure 1. The model and structure of this review used in this article are inspired by the works of Gagan Deep Sharma et al. (2020).

## Results

**FIGURE 2:**
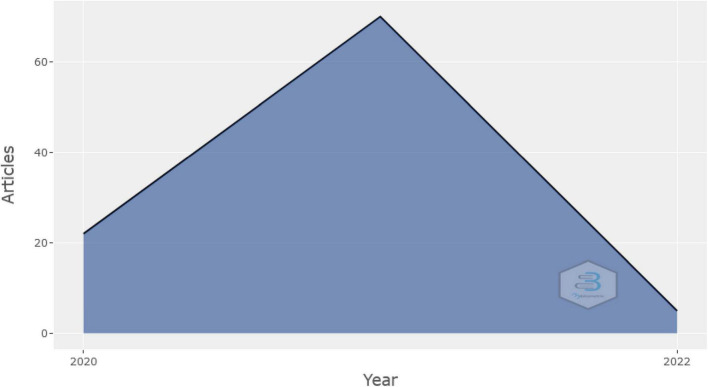
Details of year-wise publications.

This section of this article analyses the bibliometric patterns related to research on psychological linkages of financial wellbeing during COVID-19 by using the database of the Web of Science. Detailed analysis of journals, authors, countries, keywords, and research institutions is also included. The annual scientific publications, starting from the breakout of COVID-19 are shown in Figure 2. In 2020, there are twenty-two publications on this topic and seventy in the year 2021.

### Journal Analysis

**FIGURE 3:**
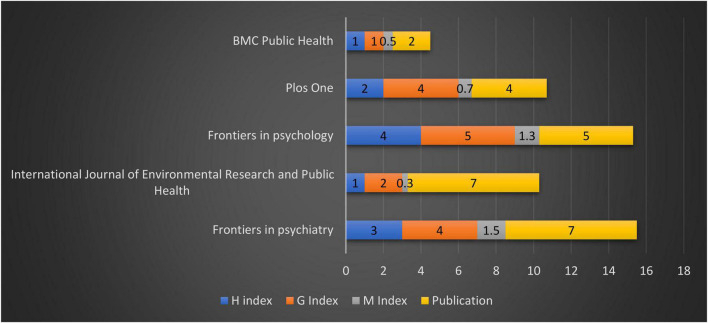
Prominent journals.

The top journals related to psychological links related to financial wellbeing during COVID-19 based on publications, H, G, and M indexes are shown in Figure 3. The top source documents are Frontiers in Psychiatry, Frontiers in Psychology, International Journal of Environmental Research and Public Health, Plos One, and BMC Public Wealth with total citations twenty-one, sixty-nine, nine, eighty, and twenty-four, respectively. The top funding agencies are the Medical Scientific Fund of the Mayor of the City of Vienna, Alberta Children Hospital Foundation, Alberta Innovates Grant, and Alberta Innovates Interdisciplinary Team Grant. The leading publishers in this topic are Frontiers Media SA with twelve publications, followed by MDPI, Public Library Science, and Springer Nature.

### Author Analysis

**FIGURE 4:**
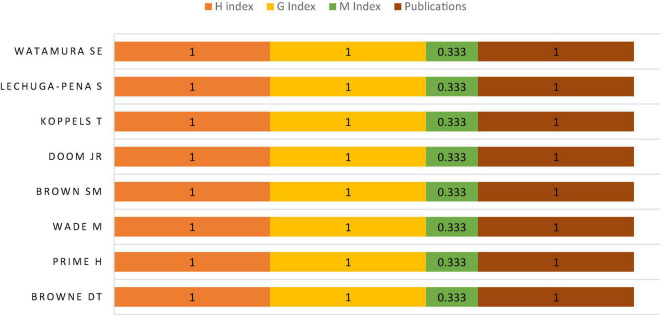
Leading authors.

The leading authors are Watamura SE, Lechuga-Pena S, Koppels T, Doom JR, Brown SM, Wade M, Prime H, and Browne DT. The H index and G index of all these authors are one. The M index of all these authors is 0.3 and all these authors have one publication each. Wade M of the University of Toronto, Prime H of York University, and Browne DT of the University of Waterloo are the leading authors in citations. All these three authors have four hundred and nineteen citations each for a collaborated paper related to the financial insecurity and psychological challenges among families during COVID-19. The other leading authors in citations are Watamura SE, Lechuga-Pena S, Koppels T, Doom JR, and Brown SM have two hundred and fifty citations each. The leading authors based on citations are shown in Figure 4 and it also shows respective the H index, G index, M index, and the number of publications by the leading authors.

### Country Analysis

**FIGURE 5:**
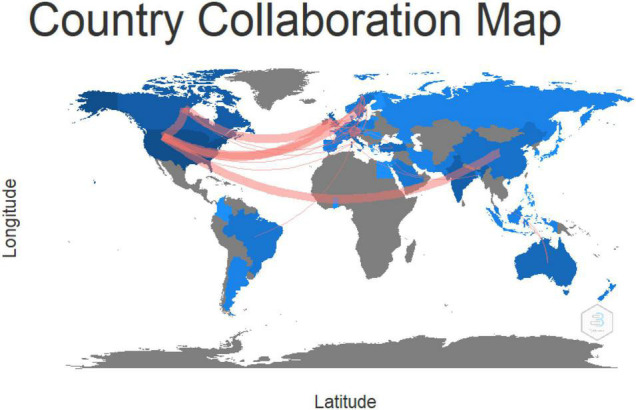
Collaboration between countries.

The collaboration between countries in the research on psychological links with financial wellbeing during COVID-19 is shown in Figure 5. The red line indicates the country collaborations and the thickness of the line depicts the strength. The leading countries are Canada, China, India, Australia, the European Union, and the United States of America, shown in dark blue color. Very strong collaboration exists among these leading countries.

Table 1 shows that the United States of America leads the research domain with thirty-six publications and twenty of them are funded documents. The country has five hundred and forty-six citations also. The United States of America is followed by Canada with fifteen document publications and has nine funded papers. The other leading papers countries are India and England.

**TABLE 1:** Country analysis.

Country	Publication	Citation	Funded document	H-index
United States	36	546	20	9
Canada	15	525	9	7
India	13	41	4	3
England	10	49	10	4

**FIGURE 6:**
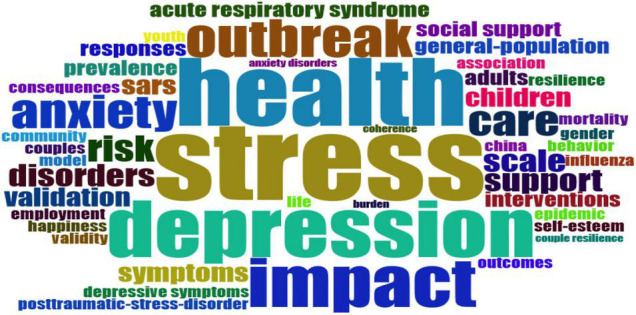
Keyword analysis.

The most occurred keywords used are shown in Figure 6.

The leading research institutions engaged in the research on psychological links related to financial wellbeing during COVID-19 are shown in Table 2.

**TABLE 2:** Leading research organizations.

Organization	Publication
Medical University of Vienna, Austria	12
University of Otago, New Zealand	11
University of Toronto, Canada	11
Royal Melbourne Hospital, Australia	10
University of Calgary, Canada	10

**FIGURE 7:**
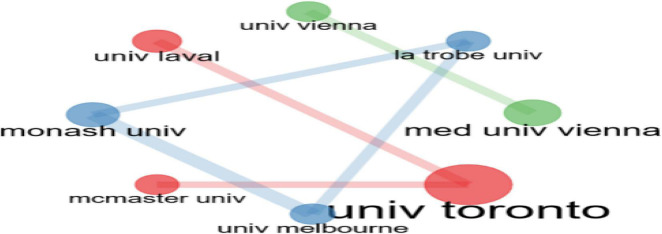
Collaboration among research institutions.

The collaboration among research affiliations is shown in Figure 7. Very strong research collaboration in this topic among the University of Toronto, University of Laval, and Mc master University; and among the University of Melbourne, La Trobe University, and Monash University. Similarly, strong research collaboration on psychological links related to financial wellbeing during COVID-19 exists among the Medical University of Vienna and the University of Vienna.

## Thematic Analysis

**FIGURE 8:**
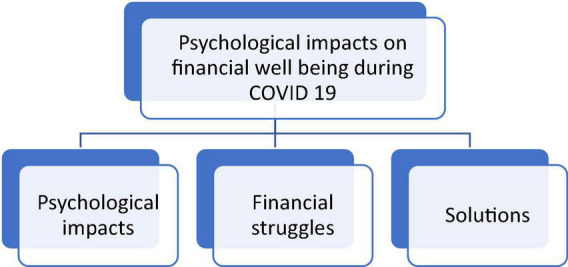
Key themes discussed in thematic analysis.

This article focuses strictly on the three major themes of psychological impacts associated with financial wellbeing during COVID-19, and the financial struggles and stress related to financial wellbeing during COVID-19 are shown in Figure 8.

### Psychological Impacts

The mental health of the financially struggled people has been deeply affected during the pandemic and the mental wellbeing of such people needs to be recovered, for copying them to lead the future life successfully (Bahar Moni et al., 2021). The mental health of the financially struggling population are affected during the COVID-19 and the healthcare needs were negatively affected, especially the people who are outside the health insurance coverage (Abufaraj et al., 2021). Nursing staff expects more rewards as compensation for their heavy workload (Akkuş et al., 2021); anxiety on the financial liabilities among orthopedic surgeons (Attarde et al., 2021); anxiety related to financial wellbeing during COVID-19 among the otorhinolaryngologists (Dosemane et al., 2021); dentists (Kumar et al., 2021) works as psychological stress in certain cases.

The psychological stress of the people who faced discrimination, poverty, and racism during their pre-COVID-19 periods is very severe during the pandemic (Asmundson et al., 2020). Parental psychology needs much attention, especially in the case of parents with unstable incomes (Fontanesi et al., 2020; Low and Mounts, 2022) and parents from the small income categories (Cheng et al., 2021; Economou, 2021; Kerr et al., 2021). Students are facing stress due to financial difficulties during pandemics like COVID-19 (Galanza et al., 2021). Gambling tendencies are another important variable that can create serious psychological pressures due to the concerns of financial challenges during the COVID-19 (Hakansson et al., 2020; Swanton et al., 2021). Attention should be paid to the psychological stress due to educational disparities, resulting due to financial distress during COVID-19 (Jiang et al., 2021).

People with mental and financial stress may react differently, like with psychological distress, anxiety, poor wellbeing, alcohol consumption, and suicidal thoughts (Bell et al., 2021). Psychological stress is also experienced in people during COVID-19 periods, other than the stress from financial challenges, the career change intentions and pressures of a workforce reduction are putting shadows on the financial wellbeing (Chen and Chen, 2021). The depression due to financial distress can lead to depression and ultimately to social problems of joblessness, divorce, living in a joint family, food scarcity, excessive sleep, and smoking (Islam et al., 2021; Siddique et al., 2021).

The financial better than average effect has impacted many people in their perspective to the near economic conditions of home, nation, and world. These people consider that their financial wellbeing is not much affected during the pandemic. Their psychological attitude toward the financial wellbeing of the home, nation, and world proves the effect of this variable and points to another dimension of the people’s psychology toward the financial wellbeing during COVID-19 (Barrafrem et al., 2020).

### Financial Struggles

Global financial conditions were affected by the pandemic and so the case of individuals. The impacts of COVID-related financial distress have effects on mental health and the economy. Millions were added to the list of poverty and hunger. Reduced family incomes are the primary cause of the financial struggles associated with COVID-19 (Islam et al., 2021). The empirical studies in Austria and Turkey (Akkaya-Kalayci et al., 2020); the United Arab Emirates (Cheikh Ismail et al., 2021); Switzerland (Marmet et al., 2021); and New Zealand (Bell et al., 2021) support the fact that people financially struggled during the COVID-19. It was also observed that the people faced psychological stress related to financial security, future, and work-life balance (Asmundson et al., 2020).

A study on Jordan related to the gender disparities and healthcare facilities during COVID-19 has found that the limited coverage of the health insurance coverage, reduced payments, denial of payment to women during the COVID-19, and job losses affected the financial wellbeing and the pregnant women were unable to undertake the antenatal care (Abufaraj et al., 2021; Mecik et al., 2022; Stevenson et al., 2022). The financial challenges of pregnant women during COVID-19 were also identified in the study (Johnson, 2021). Child care is another important segment affected by the financial distress by COVID-19 (Vance et al., 2021). The findings from Poland hint that the financial insecurity during COVID-19 and related psychological problems has resulted in the postponement of childbearing intentions (Kaye et al., 2021; Malicka et al., 2021; Raymond and Ward, 2021).

The financial struggle among healthcare workers, especially the nursing staff has the opinion of better compensation during a pandemic due to their heavy workload (Akkuş et al., 2021; Cho et al., 2021). Similarly, the financial liabilities was a major concern for the orthopedic surgeons during the COVID-19 (Attarde et al., 2021); the quality of life and financial implications of COVID-19 among the otorhinolaryngologists (Dosemane et al., 2021); and financial security concern among dentists (Kumar et al., 2021).

Researchers have also found that job insecurity causes addictive behavior and financial insecurity promotes personal conflicts (Chindarkar et al., 2021). The COVID-19 is also increasing the stress level of the parents with unstable incomes (Fontanesi et al., 2020) and parents from small income setups (Kerr et al., 2021). Moreover, educational disparities can also happen due to financial distress during pandemic (Jiang et al., 2021). Students are another vulnerable community to financial stress during COVID-19 (Galanza et al., 2021; Schröpfer et al., 2021; Kokkinos et al., 2022) and impacted by the economic instability and psychological issues (Li et al., 2021); old-aged community (Heid et al., 2021; Kivi et al., 2021). However, there are contradicting findings that the dental students are least concerned about their financial status during pandemic (Poma et al., 2021). Several studies have also reported that certain sections of the societies have comparatively less psychological impacts due to the stress on financial wellbeing during COVID-19 (Barrafrem et al., 2020; Chiaravalloti et al., 2021).

### Solutions for Reducing Psychological Stress Related to Financial Wellbeings

There cannot be a one-time solution for all psychological pressures and all these psychological stresses cannot be handled similarly to all the financial stresses experienced during COVID-19. The psychological issues related to underpaying and overwork of nursing staff can be solved by developing better packages during pandemics (Akkuş et al., 2021). The financial struggles of vulnerable sections through financial support (Hahlweg et al., 2020); financial support or assistance through social security schemes can alleviate the financial and psychological distress due to the COVID-19 (Jha et al., 2021). The challenges for the healthcare facilities due to financial issues in COVID can be through better healthcare coverages (Abufaraj et al., 2021); publicly funded healthcare systems (Letourneau et al., 2021); and financial support for pregnant women (Johnson, 2021). Promoting the psychological health of entrepreneurs (St-Jean and Tremblay, 2021).

Spiritual wellbeing and social support are other alternatives to cope with the COVID-19-induced financial distress and related psychological stress (Nia et al., 2021). The proper financial planning can be a good measure to face financial crisis and manage the finance-related psychological pressure during a pandemic for the pre-elderly groups, but may not be working well with elders (Ooi et al., 2021). Policy interventions are very essential for solving the financial and health challenges of older people during the pandemic (Pothisiri and Vicerra, 2021).

Mentorship can be an alternative to coping with psychological challenges due to financial distress during pandemics among low-income students. Such innovative interventions can enhance mental strength among financially vulnerable students during pandemics (Lee et al., 2021). University support to the student community can help to reduce the psychological challenge related to financial distress during pandemics (Poma et al., 2021) and long-term initiatives for supporting students and improving their mental health (Schmits et al., 2021).

## Future Research Agenda

**FIGURE 9:**
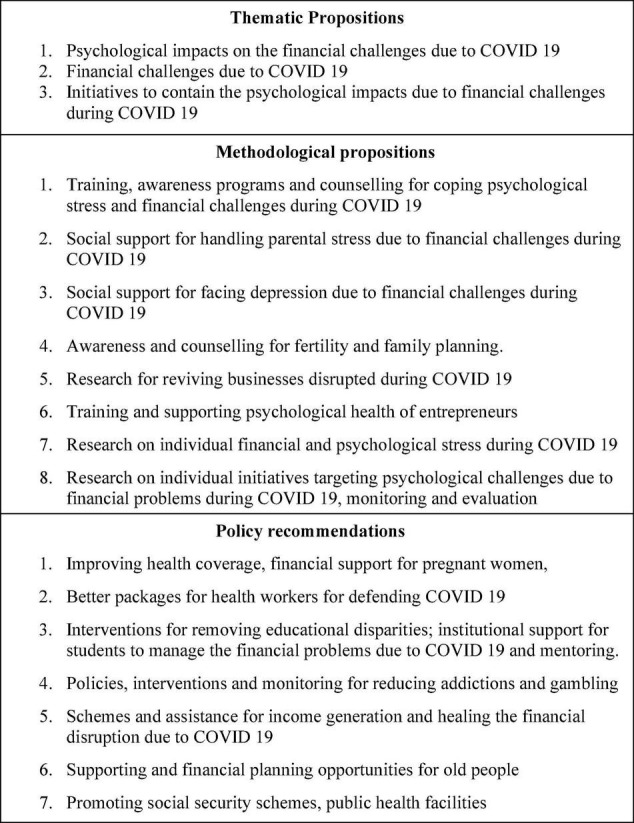
Agenda for future research.

The following propositions are developed by the detailed thematic analysis of the literature related to the psychological underpinning of financial wellbeing. The scope for future research is also included in this section below in the Figure 9.

### Thematic Propositions

Future researchers can concentrate on the three core themes identified by this review for research on the psychological impacts of financial wellbeing during COVID-19. The major themes can be psychological impacts on the financial challenges due to COVID-19; various financial challenges due to COVID-19; and finally the initiatives to contain the psychological impacts due to financial challenges during COVID-19.

### Methodological Propositions

Future researchers can focus on providing training, arranging awareness programs, and counseling for coping with psychological stress and financial challenges during COVID-19. These initiatives can bring many people out of poverty and hunger. Parental stress due to loss or low or unstable income should be reduced by providing social support for reducing their stress and recovering their earning potentials. Social support can also provide for the needy ones facing depression and related psychological impacts due to psychological stress related to financial problems during COVID-19. Awareness and counseling programs for people who have avoided and postponed childbirth initiatives can be facilitated along with concepts of family planning and fertility. Business revivals can be good empirical themes for future research in the post-COVID period. Training and support for improving the psychological health and strength of entrepreneurs can also be meaningful topics for future research. Empirical or reviews on each psychological stress factor and financial challenge faced during COVID-19 can be promising themes for future research. The research on individual initiatives for coping with psychological pressure due to financial problems during COVID-19, monitoring of the initiatives, and performance appraisal can also be good niches for future research.

### Policy Propositions

Various policy initiatives are being recommended after the detailed literature review and thematic analysis on the research related to psychological stress due to financial challenges faced during COVID-19. The policies propositions related to this topic can be for taking initiatives and policies for speeding up the health insurance coverage and speeding up the financial support for all pregnant women and children. Better packages can be tailored for the healthcare workers for their efforts to contain the pandemic. COVID-19 has disrupted the education sector. This article recommends research and policies for removing educational disparities due to financial distress during COVID-19. Institutional support and mentoring should be provided for students vulnerable to financial problems during COVID-19. Policies, interventions, and monitoring should be initiated for controlling addictions and gambling. Schemes should be developed for providing assistance for income generation and healing the financial disruptions due to COVID-19. Old-aged people have poor financial planning and are vulnerable to financial problems and psychological stress during COVID-19. Support systems and financial planning opportunities for the old-age people for their welfare during COVID-19. Strong policies should be developed for rolling out the social security schemes, and for ensuring quality public health facilities. The policies need to be human-centric rather than focused on economic recovery because COVID-19 not just shook the economies but it is a greater challenge for the entire human community.

## Conclusion

The major financial challenges for the financial wellbeing during COVID-19 are the impacts on the income of the people (Islam et al., 2021), impacts on financial security, future, and work-life balance (Asmundson et al., 2020) due to reduced jobs and job losses and low-level salaries. Poverty and hunger have increased all over the world. The education sector is highly vulnerable to a pandemic. The education disparities (Jiang et al., 2021) and financial challenges of students (Galanza et al., 2021; Li et al., 2021; Schröpfer et al., 2021; Kokkinos et al., 2022) are important issues to be addressed and institutional support (Poma et al., 2021; Schmits et al., 2021) and mentoring (Lee et al., 2021) can be a good move in this regard.

The poor coverage of health insurance and financial problems forced people to postpone or avoid the institutional services related to pregnancy antenatal care (Abufaraj et al., 2021; Johnson, 2021), child care (Vance et al., 2021), and postponement of childbearing intentions (Malicka et al., 2021). Rapid coverage of health insurance policies (Abufaraj et al., 2021), counseling for childbearing and family planning, social security schemes, financial support for pregnant women (Johnson, 2021), and neonatal care and improving public health facilities (Letourneau et al., 2021) are some health-related measures to cope with these challenges. Healthcare workers struggled for better packages matching their efforts for defending COVID-19, especially nurses (Akkuş et al., 2021; Cho et al., 2021); orthopedic surgeons (Attarde et al., 2021); otorhinolaryngologists (Dosemane et al., 2021); and dentists (Kumar et al., 2021). Better packages are recommended for the issues of healthcare providers (Akkuş et al., 2021).

The psychological pressures of parents with unstable incomes (Fontanesi et al., 2020) and small income setups (Kerr et al., 2021) have been a big concern during the pandemic. Similarly, the psychological and financial challenges of the old-aged community (Heid et al., 2021; Kivi et al., 2021). Social support for vulnerable parents and old-aged people along with financial planning can reduce the sufferings of this section (Pothisiri and Vicerra, 2021). The other initiatives for managing the psychological impacts of financial challenges during COVID can be spiritual lessons (Nia et al., 2021). This article also recommends future research for handling parental stress, facing depression, reviving disrupted business, fostering income and employment, and training entrepreneurs. The recommendations are also for fresh initiatives related to reducing gambling and addiction.

Very few research interests have been on this topic, around 115 document publications, namely, articles, reviews, and conference papers. The United States of America leads the domain in document publications and citations and followed by Canada. Very few research collaborations exist among countries. However, there are strong collaborations among authors and institutions. This article is used for identifying the financial challenges, identifying the vulnerable communities, sorting out the psychological challenges, and developing policy interventions. This article can be useful for future research scholars and academicians for future research. Similarly, it will be easy for policymakers and administrators for focusing on the problem, measure the dimensions of impact and for implementing solutions. This article can also be useful for finding out the leading researchers, most occurred keywords, and country collaborating research on this topic. Moreover, the leading sponsors and research institutes can be identified along with the research affiliations engaged in the collaborated research.

The three major funding agencies on this topic are the National Institutes of Health of the United States of America, the United States Department of Health Human Services, and the Canadian Institutes of Health Research. National Institutes of Health Research and the United States Department of Health Human Services have five funded projects on this topic with 367 citations. The topics of these funded projects include psychological wellbeing (Wanberg et al., 2020); gambling and COVID crisis (Hakansson et al., 2020); stress and parenting during COVID-19 (Brown et al., 2020); challenges of old-aged people during COVID-19 (Heid et al., 2021); and related to the COVID impact on caregivers of childhood cancer survivors (Wimberly et al., 2021). Canadian Institutes of Health Research has four funded papers on this topic with 252 citations. The funded papers were on the topic of financial and psychological impact to mothers and children during COVID-19 (Letourneau et al., 2021); Chinese Canadians’ experience during pandemic (Lou et al., 2021); and related to sleep-related issues and psychological impacts during COVID-19 (Morin et al., 2020, 2022). The pandemic opened new areas for the researcher to work upon the goals of the human community. There is a need to study human psychology and human needs in more depth as we have seen that COVID-19 created a huge disruption not only economically but also mentally.

## Data Availability Statement

Publicly available datasets were analyzed in this study. This data can be found here: https://www.scopus.com/ and https://www.webofscience.com/.

## Author Contributions

SS conceptualized the idea, wrote the introduction, and methodology. DB collected the literature and wrote the conclusion. Both authors contributed to the article and approved the submitted version.

## Conflict of Interest

The authors declare that the research was conducted in the absence of any commercial or financial relationships that could be construed as a potential conflict of interest.

## Publisher’s Note

All claims expressed in this article are solely those of the authors and do not necessarily represent those of their affiliated organizations, or those of the publisher, the editors and the reviewers. Any product that may be evaluated in this article, or claim that may be made by its manufacturer, is not guaranteed or endorsed by the publisher.
